# MAPPING THE FACTORS OF LONELINESS IN OLDER ADULTS: THE ROLE OF EMPLOYMENT AND WORK ACTIVITY – A SCOPING REVIEW

**DOI:** 10.13075/ijomeh.1896.02722

**Published:** 2025

**Authors:** Katarzyna Domosławska-Żylińska, Magdalena Łopatek, Dominik Olejniczak

**Affiliations:** 1 National Institute of Public Health NIH – National Research Institute, Department of Health Promotion and Prevention of Chronic Diseases, Warsaw, Poland; 2 Medical University of Warsaw, Division of Public Health, Faculty of Health Science, Warsaw, Poland

**Keywords:** employment, retirement, loneliness, older adults, healthy ageing, social isolation

## Abstract

Loneliness and social isolation are increasingly recognized as major public health challenges, particularly among older adults. This review aims to identify and map the key risk and protective factors associated with loneliness and social isolation, with particular emphasis on the role of employment and work activity in later life. In accordance with Preferred Reporting Items for Systematic Reviews and Meta-Analyses extension for Scoping Reviews (PRISMA-ScR) guidelines, peer-reviewed studies published in 2019–2025 in the PubMed and Scopus databases were analyzed. The results showed that health-related, psychological, sociocultural, and socio-environmental factors are closely associated with the experience of loneliness in old age. The most numerous were health-related and sociocultural factors. Employment and social engagement played a particularly important role in mitigating loneliness by providing structure, purpose, and social contact. The transition from work to retirement was identified as a critical life stage that can either intensify or alleviate loneliness, depending on individual circumstances and the cultural context. Future research should adopt a longitudinal and cross-cultural approach to better understand how the moment of retirement affects loneliness in the long term. There is also a need to explore gender and socioeconomic differences in how older adults experience and cope with social isolation. Moreover, evaluating the effectiveness of workplace and community-based interventions aimed at fostering social connectedness after retirement could provide valuable evidence for developing public health strategies that support healthy and active ageing.

## Highlights

- Health and sociocultural factors dominate loneliness in later life.
- Strong social ties are the best protection against loneliness and social isolation.
- Retirement – a turning point: does it deepen or ease loneliness?

## INTRODUCTION

Loneliness and social isolation are increasingly recognized as significant public health challenges, particularly affecting the ageing population worldwide [1]. The number of individuals aged ≥60 years in the WHO European Region is rapidly increasing – from 215 million in 2021 to an estimated 247 million by 2030, and >300 million by 2050 [2]. It is estimated that 20–34% of older adults in Europe experience loneliness or social isolation [3]. In the scientific literature, the concepts of loneliness and social isolation are often analyzed together, even though they refer to different phenomena. However, both are interrelated and can have a comparably adverse effect on an individual's health [4]. Social isolation refers to an objective lack of social contacts, whereas loneliness is a subjective experience of feeling alone, regardless of the actual number of social relationships. In other words, while isolation means an actual reduction in social interaction, loneliness reflects a perceived deficit in this area.

It is important to note that a limited social network or frequent solitude are not necessarily experienced negatively whereas feelings of loneliness may arise even in the presence of an sufficient number of interpersonal contacts [5–8]. In addition, social and emotional loneliness can be distinguished. Social loneliness is characterized by a lack of a satisfying social network and a perceived sense of rejection, while emotional loneliness is a lack of a close, committed relationship, such as an intimate partner [9]. Loneliness and social isolation are recognized as risk factors for both mental and physical health. They are associated with, among other things, a 50% increase in the risk of developing dementia, an increased risk of depression, anxiety, diabetes, cancer, a 30% increase in the risk of coronary heart disease or stroke, and a 26% increase in the risk of death from any cause. The adverse effects of loneliness lead to increased use of health and social services, which has a growing impact on social costs [4,10]. Risk and protective factors associated with social isolation and loneliness can be categorized into: health factors (e.g., chronic diseases, functional impairments), psychological and cognitive (e.g., depression, anxiety, dementia), sociocultural (e.g., social support, destructive life events), and socio-environmental (e.g., transportation, housing) [11].

Older adults are particularly vulnerable to experiencing loneliness due to the weakening of social ties resulting from significant life transitions, such as retirement, the loss of loved ones, reduced mobility, or declining functional capacity associated with chronic illness or disability [4]. Retirement is a significant turning point in an individual's life cycle, associated with the end of professional activity, loss of regular income from work, and the definitive end of a professional career. The literature emphasizes that this phase is often associated with increased stress levels and the breakdown of existing social and professional roles. It is a critical time when the loss of structured social interactions and emotional support can lead to increased loneliness [12]. Retirement circumstances can be categorized into 2 main types: voluntary and compulsory. Voluntary retirement is based on an individual's personal decision to end professional activity, regardless of whether this occurs before or after reaching the statutory retirement age. Compulsory retirement, on the other hand, occurs as a result of external factors beyond the individual's control. Research shows that compulsory retirement can have a negative impact on mental health, leading, among other things, to reduced life satisfaction, higher stress levels, and an increased risk of depressive symptoms [13]. Employment plays an important role in maintaining social engagement and providing social support. Therefore, retirement involves significant changes in an individual's lifestyle and social environment.

Older people who spend most of their time at home – for example, due to mobility limitations, health conditions, or lack of support – may find it difficult to maintain social relationships. These problems are particularly evident in situations where access to public transport is limited or digital literacy is low, making it difficult to establish and maintain social contacts and take advantage of available forms of social support [14]. Therefore, it is necessary to deepen the understanding of the relationship between employment status and loneliness in later life, which may be an important step towards a better understanding of the psychosocial needs of the aging population. The aim of the present study is to identify and map key risk and protective factors associated with social isolation and loneliness, and to examine the relationship between employment status and experiences of loneliness and social isolation among older adults.

## METHODS

This study used the scoping review methodology, in accordance with the guidelines in Preferred Reporting Items for Systematic Reviews and Meta-Analyses extension for Scoping Reviews (PRISMA-ScR). The review aimed to gather synthetic information and map the risk factors for loneliness among older adults, with particular emphasis on the role of employment and retirement. The review was conducted in September 15 – October 3, 2025. The literature search was conducted in the PubMed and Scopus databases. The following keywords were used: older adults or elderly or seniors; loneliness, social isolation, risk factors or predictors or determinants; job or employment or retirement or work activity. The core search query was structured as follows: (“older adults” OR elderly OR seniors) AND (loneliness OR “social isolation”) AND (“risk factors” OR predictors OR determinants).

The review included articles that: were published in peer-reviewed scientific journals in 2019–2025, were available in full text, in English or Polish, presented research results concerning older people (most often defined as people aged ≥60 years), examined loneliness and/or social isolation, identified risk factors or predictors of these phenomena, including in particular those related to employment or retirement. The following were excluded from the analysis: articles that did not concern the older population, non-empirical publications (e.g., commentaries, essays, letters to the editor), works without access to the full text, studies not related to loneliness or social isolation, articles focusing exclusively on interventions, without analysis of risk factors.

### Study selection and data extraction

**Figure 1.**
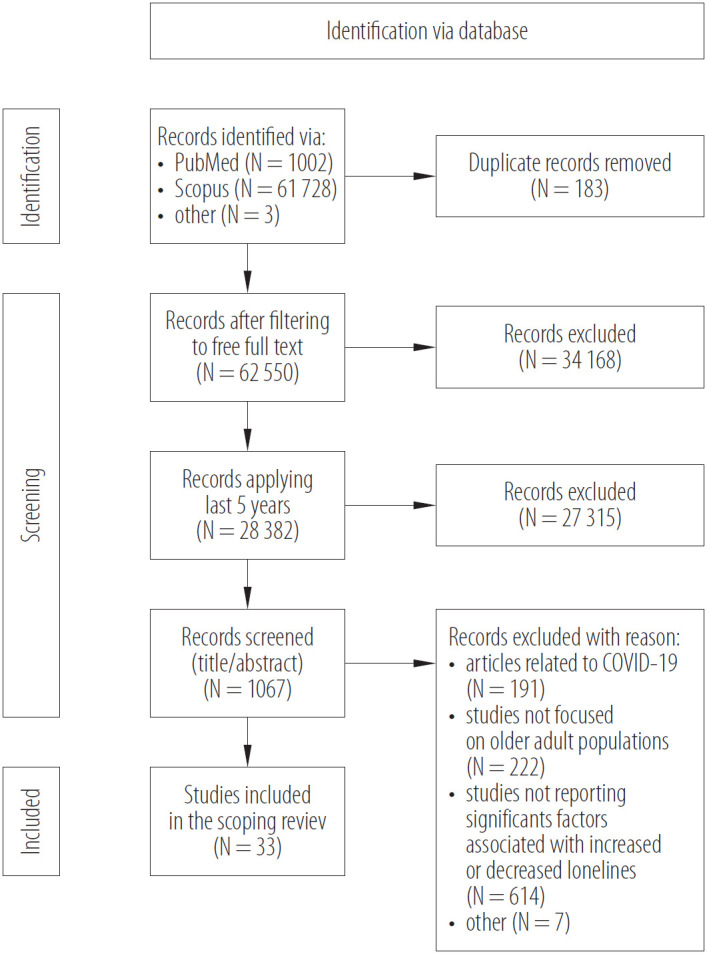
PRISMA – Scoping Review (ScR) extension flow diagram of the identification process for the sample of 33 articles describing factors of loneliness and social isolation in older adults included in this review on factors of loneliness in older adults, September 15 – October 3, 2025

In the initial screening phase, 2 independent reviewers assessed titles and abstracts of all retrieved records to identify potentially relevant studies. Discrepancies were resolved through discussion or consultation with a third reviewer when necessary. Subsequently, the full texts of potentially eligible articles were independently reviewed against predefined inclusion and exclusion criteria to determine final eligibility. Data extraction was performed independently by both reviewers using a standardized data extraction form developed for this review. Extracted information included study characteristics (authors, year, study design), identified risk and protective factors. A total of 33 articles were included in the final analysis (Figure 1). The results were presented according to the main groups of factors: health, psychological, sociocultural, and socio-environmental. The collected data were analyzed using narrative synthesis, where factors related to professional activity were identified.

A total of 62 733 records were identified through database searches (PubMed N = 1002, Scopus N = 61 728, other N = 3). After removing 183 duplicates, 62 550 records remained and were screened for free full-text availability. Following the exclusion of 34 168 records and limiting the search to the last 5 years, 28 382 records were retained. Subsequent screening of titles and abstracts resulted in the exclusion of 27 315 studies. The remaining 1067 articles were evaluated in detail, of which 33 studies met the inclusion criteria and were included in the final scoping review.

The most frequent reasons for exclusion were: articles related to COVID-19 (N = 191), studies not focused on older adult populations (N = 222), studies not reporting significant factors associated with increased or decreased loneliness (N = 614), and other reasons (N = 7) (Figure 1).

## RESULTS

A total of 33 studies met the inclusion criteria and were included in the final scoping review. The included studies varied in design, methodology, and geographic scope, providing a comprehensive overview of the determinants of loneliness among older adults. The majority of the research was conducted in Europe with study designs encompassing systematic reviews, meta-analyses, cross-sectional studies, longitudinal cohort studies, and scoping reviews. A detailed summary of the included studies, including the source database, authors, study type, and identified factors (Table 1)

**Table 1.** Articles included in the scoping review on factors of loneliness in older adults with emphasis on the role of the role of employment and work activity, published in peer-reviewed scientific journals in 2019–2025, review conducted in September 15 – October 3, 2025

Reference	Database	Title	Study type	Factors	
				increasing loneliness	reducing loneliness
Kolk et al. [15]	PubMed	A scoping review of influencing factors associated with loneliness in nursing home settings	scoping review	prevalence of chronic diseases (cardiovascular system, chronic lower back pain, rheumatoid arthritis, diabetes), poor physical fitness, loss of spouse/no partner	more frequent social contacts
Hajek et al. [16]	PubMed	Chronic loneliness and chronic social isolation among older adults. A systematic review, meta-analysis and meta-regression	systematic review, meta-analysis and meta-regression	living alone	more frequent social contacts, living with a partner
Liu et al. [17]	Scopus	Combined association of multiple chronic diseases and social isolation with the functional disability after stroke in elderly patients: a multicenter cross-sectional study in China	cross-sectional	prevalence of chronic diseases: cardiovascular system, chronic lower back pain, rheumatoid arthritis, diabetes, presence of pain, multimorbidity, post-stroke condition, disability, smoking and alcohol consumption	membership in an organization
Gough et al. [18]	PubMed	Community participation of community dwelling older adults: a cross-sectional study	cross-sectional	lack of physical contact	physical contact
Fancourt and Tymoszuk [19]	Scopus	Cultural engagement and incident depression in older adults: evidence from the English Longitudinal Study of Ageing	longitudinal cohort study	loss of spouse/no partner	cultural involvement and activity (visiting museums/galleries/exhibitions, going to the theater/concerts/opera, but not to the cinema
Hajek et al. [20]	PubMed	Determinants of loneliness among older adults: a systematic review based on recent longitudinal studies	systematic review (longitudinal)	poor self-assessment of health, having a sense of meaning/purpose in life, high agreeableness, owning a cat	marital status, volunteering, cohesive/integrated/neighborhood, strong ties to the local community
Jutengren and Ståhl [21]	Scopus	Determinants of social loneliness among older adults in job retirement and the role of emotional expressivity	cross-sectional study	retirement	extroversion
Björnwall et al. [22]	PubMed	Eating alone or together among community-living older people – a scoping review	scoping review	eating alone, lack of physical contact	shared meals, social participation
Balki et al. [23]	PubMed	Effectiveness of technology interventions in addressing social isolation, connectedness, and loneliness in older adults: systematic umbrella review	umbrella review	digital barriers	use of digital technology to enhance social connectedness
Smale et al. [24]	Scopus	Exploring the determinants and mitigating factors of loneliness among older adults	cross-sectional study	lack of contact with children, relatives, and friends; living alone; difficult access to transportation	
Juma et al. [25]	Scopus	Intensity and changes in grandparental caregiving: exploring the link to loneliness in Europe	quantitative longitudinal study	starting to care for grandchildren	stable caregiving role for grandchildren
Hoang et al. [26]	PubMed	Interventions associated with reduced loneliness and social isolation in older adults: a systematic review and meta-analysis	systematic review and meta-analysis	health-related	animal-assisted therapy, social/technological interventions, support, engagement, housing conditions
Tcymbal et al. [27]	PubMed	Interventions simultaneously promoting social participation and physical activity in community living older adults: a systematic review	systematic review (interventions)		physical activity
Tully et al. [28]	Scopus	Is sedentary behavior or physical activity associated with loneliness in older adults? Results of the European-wide SITLESS study	cross-sectional study	lack of physical activity	physical activity
Pollak et al. [29]	PubMed	Loneliness and functional decline in aging: a systematic review	systematic review	functional decline	social activity
Greš et al. [12]	other	Loneliness in retirement	narrative review	retirement	
Pollak et al. [30]	Scopus	Loneliness predicts progression of frailty in married and widowed, but not unmarried community dwelling older adults	longitudinal study	high level of frailty, loss of spouse/no partner	marital status
Nakou et al. [31]	PubMed	Loneliness, social isolation, and living alone: a comprehensive systematic review, meta-analysis, and meta-regression of mortality risks in older adults	systematic review, meta-analysis and meta-regression	loss or decline in cognitive function, older age, female gender, lower education	volunteering
Schutter et al. [32]	PubMed	Loneliness, social network size and mortality in older adults: a meta-analysis	meta-analysis	decrease in social network size	
Sipowicz et al. [33]	Scopus	Lonely in the city – sociodempographic status and somatic morbidities as predictors of loneliness and depression among seniors – preliminary results	cross-sectional study	depression	
Hounkpatin et al. [34]	PubMed	Multiple long-term conditions, loneliness and social isolation: a scoping review of recent quantitative studies	scoping review	presence of pain, multimorbidity	
Bevilacqua et al. [35]	other	Older working adults in the HEAF study are more likely to report loneliness after 2 years of follow-up if they have negative perceptions of their work quality		sleep problems, renting an apartment (rather than owning one)	instrumental support
Sugiura et al. [1]	other	Relationship between employment status, loneliness, and social isolation: a systematic review	systematic review	retirement	having a job
Sutin et al. [36]	PubMed	Sense of purpose in life and concurrent loneliness and risk of incident loneliness: an individual-participant meta-analysis of 135 227 individuals from 36 cohorts	individual-participant meta-analysis		sense of purpose
Resna et al. [37]	PubMed	Social environment support to overcome loneliness among older adults: a scoping review	scoping review		membership in an organization, support from family and friends
Gallardo-Peralta et al. [38]	PubMed	Studying loneliness and social support networks among older people: a systematic review in Europe	systematic review	loss of spouse/no partner	support from family and friends
Hreha et al. [39]	Scopus	The association of vision and hearing impairment on cognitive function and loneliness: evidence from the Mexican health and aging study	cross-sectional study		membership in an organization
Qorbani et al. [40]	PubMed	The effect of participation in support groups on retirement syndrome in older adults	quasi-experimental		participation in support groups
Rodrigues and Delerue-Matos [41]	PubMed	The effect of social exclusion on the cognitive health of middle-aged and older adults: a systematic review	systematic review	social exclusion	
Salari et al. [42]	Scopus	The global prevalence and associated factors of loneliness in older adults: a systematic review and meta-analysis	systematic review and meta-analysis	experience of falls and/or fear of falling, use of painkillers and antidepressants, sleep problems, depression, high levels of neuroticism, informal caregiving	better sleep quality, high level of health literacy, positive assessment of quality of life (mental and physical)
Robinson et al. [43]	Scopus	The psychological burden associated with metabolic syndrome: evidence from UK and US older adults	cross-sectional study	obesity	
Puyané et al. [44]	PubMed	Uncovering the impact of loneliness in ageing populations: a comprehensive scoping review	scoping review	loss of social contacts, chronic or mental diseases	social support, social participation
Band and Rogers [45]	PubMed	Understanding the meaning of loneliness and social engagement for the workings of a social network intervention connecting people to resources and valued activities	qualitative (process evaluation)	difficult access to transportation	

An analysis of the identified studies allowed for the identification of a wide range of factors influencing the experience of loneliness and social isolation among older people. A total of 50 risk factors and 25 mitigating factors were identified, grouped into 4 main categories: health, psychological-cognitive, sociocultural and socio-environmental (Table 2). The most numerous group consisted of health and sociocultural factors, which indicates the key role of both physical health and social environment in shaping the level of loneliness in later life. Analysis of the identified studies allowed for the identification of a wide range of factors influencing the experience of loneliness and social isolation among older people. In the area of health factors, variables related to deteriorating health and limited physical fitness dominated, including the presence of chronic diseases, pain or disability. On the other hand, mitigating factors in this group included physical activity, good self-rated health and high health awareness. This indicates that maintaining activity and good functional status can play an important protective role against loneliness. Among psychological and cognitive factors, personality traits (e.g., high neuroticism, low conscientiousness) and the presence of depressive symptoms were of key importance, while factors such as extroversion, sense of meaning in life, and positive assessment of quality of life were associated with lower levels of loneliness. These results confirm the importance of mental well-being and personality in the context of social relationships in older age.

**Table 2.** Categorization of risk and protective factors associated with social isolation and loneliness across 4 domains in the scoping review on factors of loneliness in older adults, September 15 – October 3, 2025 [based on 1, 15–45]

Factors	Loneliness and social isolation	
	risk factors	protective factors
Health factors	– poor self-assessment of health – prevalence of chronic diseases: cardiovascular system, chronic lower back pain, rheumatoid arthritis, diabetes – poor physical fitness – presence of pain – obesity – multimorbidity – vision and hearing problems – post-stroke condition – disability – high level of frailty – experience of falls and/or fear of falling – smoking and alcohol consumption – use of painkillers and antidepressants – sleep problems – low level of health literacy – malnutrition/weight loss – lack of physical activity – lack of physical contact	– performing everyday activities without difficulty – positive self-assessment of health status – better sleep quality – high level of health literacy – physical activity
Psychological and cognitive factors	– loss or decline in cognitive function – high levels of psychological stress – depression – high levels of neuroticism – having a sense of meaning/purpose in life – high agreeableness – low conscientiousness – individualism – dementia – dissatisfaction with life – sadness	– extroversion – positive assessment of quality of life (mental and physical)
Sociocultural factors	– older age – female gender – lower education – retirement – loss of income – unemployment – decrease in social network size – loss of spouse/no partner – changes in family structure (e.g., economic migration of young people and leaving older people behind) – lack of contact with children, relatives, and friends – small number of close friends – starting to care for grandchildren – informal caregiving – greater degree of social isolation – eating alone – digital barriers – owning a cat	– better socioeconomic status – level of education – marital status – identifying one's spouse as one's closest confidant – having a job – membership in an organization – sense of belonging to a community/society – more frequent social contacts – participation in recreational activities – volunteering – cultural involvement and activity (visiting museums/galleries/exhibitions, going to the theater/concerts/opera) – cohesive/integrated/neighborhood, strong ties to the local community – use of new types of media/technology – support from family and friends – communal eating – use of digital technology to enhance social connectedness
Socio-environmental factors	– living in the city – living alone – renting an apartment (rather than owning one) – difficult access to transportation	– living with a partner – instrumental support

Sociocultural factors constituted the most diverse group. Risk factors included older age, female gender, low education, retirement, loss of a partner, and limited social networks. Protective factors, on the other hand, included having a job, being married, belonging to an organization, volunteering, recreational and cultural activities, and strong ties to the local community. These results confirm that social and professional activity are important factors in counteracting loneliness. In the category of socio-environmental factors, both risk factors (living alone, lack of access to transport) and protective factors (living with a partner, instrumental support) were identified.

The aim of this review was to identify and illustrate key risk factors and protective factors associated with social isolation and loneliness, with particular emphasis on the relationship between employment status and these phenomena in the elderly population. The analysis showed that numerous and diverse factors can serve as significant predictors of loneliness and social isolation in this group. Among them, the most numerous groups are factors related to physical health and sociocultural factors. In particular, numerous chronic conditions – such as cardiovascular disease, chronic lower back pain, rheumatoid arthritis, diabetes, and obesity –

show significant links to the phenomena discussed. These relationships are often bidirectional: on the one hand, loneliness and social isolation can increase the risk of developing or worsening chronic health problems, and on the other hand, the presence of a chronic disease can contribute to social withdrawal, limiting the number and quality of interpersonal relationships or disrupting their structure. In addition, this condition can have a negative impact on physiological processes, which exacerbates both the experience of loneliness and the deterioration of health [11]. Psychological factors, such as depressive symptoms and self-rated general health, also influence the development of loneliness over time. Social isolation and loneliness are more prevalent among older adults experiencing depressive and anxiety disorders [46]. Furthermore, evidence suggests a link between individual personality traits and the experience of loneliness, highlighting the complexity and multidimensional nature of this phenomenon [47]. Among sociocultural factors, various socio-demographic factors such as gender, age, social situation, and socioeconomic status also play a significant role in the experience of loneliness and social isolation. These factors influence both the availability of social support and the ways in which individuals cope with the ageing process and major life transitions, such as retirement [47]. In this context, particular attention should be paid to factors related to employment and the transition period associated with the end of professional activity. Factors contributing to increased loneliness and social isolation include retirement, loss of income, unemployment, and a reduction in the size of one's social network. On the other hand, employment, membership in organizations, a sense of community belonging, and more frequent social contacts are associated with lower levels of loneliness. The literature on the subject indicates that retirement can be associated with a sense of loss of identity, which is an important aspect of the adaptation process during this period of life. The change in social and professional roles associated with the end of a professional career often leads to difficulties in redefining one's place in society. Research suggests that adapting to new life roles after retirement can have negative psychological effects, such as anxiety or depression, and can also contribute to a deterioration in physical condition [48]. After retirement, a significant increase in emotional loneliness is also observed. Interestingly, the difference between the 5-year period and the year before the end of one's professional career did not prove to be statistically significant, suggesting that loneliness may result primarily from the process of change itself [49]. Theories concerning the relationship between loneliness and aging focus mainly on changes in social relationships and the structure of support networks, which may be important determinants of loneliness in older age. According to these theories, loneliness tends to increase in older age, particularly as a result of a decline in close relationships and reduced availability of emotional support. These changes are often associated with the loss of life partners, retirement, and limitations in physical mobility [50]. The cultural context is also a significant factor influencing the experience of loneliness after retirement. Surprisingly, however, the opposite trend has been observed in China, where retired people felt less lonely than those who remained professionally active [48]. In traditional Chinese culture, older people rely on family support, engaging in mutual care, which can protect against feelings of isolation and loss of identity. In Western cultures, on the other hand, dependence on family support is often associated with feelings of guilt and shame, which can exacerbate the experience of loneliness among older people [51,52]. Research suggests that 1 effective adaptation strategy to retirement is increasing levels of activity, which contributes to reducing feelings of loneliness and social isolation [13]. Combining retirement with continued employment seems particularly beneficial, as it allows social interactions to be maintained. Such social contacts can not only protect against loneliness, but also correlate positively with other aspects of well-being, such as subjective quality of life, health status, and lower mortality. It has also been observed that men who continued to work after retirement were less likely to experience loneliness, while those who ceased working after retirement were more vulnerable to loneliness than those who did not work or had not yet retired [53]. Unlike voluntary retirement, which is usually planned and anticipated, forced retirement can occur suddenly and without prior preparation. In such cases, people ending their professional activity often do not have sufficient emotional or social resources to effectively adapt to their new life situation. Such sudden and unwanted changes can lead to a decrease in feelings of control and self-esteem, as well as contribute to lower life satisfaction, poorer physical and mental health, unhealthy lifestyles, and tensions in family and social relationships. All of these factors increase the risk of experiencing loneliness [54]. At the same time, it has been shown that people who, despite forced retirement, had access to strong and positive social support experienced relatively lower levels of loneliness. This indicates that social support can act as a buffer to mitigate the negative effects of sudden and unplanned termination of professional activity [54].

By identifying specific, often hidden risk factors and directly addressing these issues in healthcare practice, healthcare decision-makers can help reduce social isolation and loneliness [11]. In line with this perspective, WHO has developed maps that present scientific evidence on the effectiveness of various interventions aimed at reducing social isolation and loneliness, categorized by the level of intervention – individual, interpersonal, community, and national. Individual-focused interventions (e.g., cognitive behavioral therapy, social skills training, and psychoeducation), interpersonal interventions (cognitive behavioral therapy, social skills training, psychoeducation, healthcare support, social support), community-based interventions (group activities such as gardening, art, and physical activity; support groups, including peer support groups; neighborhood support; building age-friendly communities; participation in volunteering), and national-level interventions (public health policies promoting social cohesion and integration, public education and social awareness on social relations, neighborhood design policies, and funding for relevant research) have all been identified as potentially effective approaches [55].

### Strengths and limitations

The main strength of this review is its focused anfd timely approach to analyzing loneliness and social isolation among older adults, with a particular emphasis on the role of work and retirement. Including studies published within the last 5 years, this review provides a current perspective on the growing challenges associated with population aging. In addition, the use of a methodology based on the PRISMA-ScR guidelines ensures the systematic nature, high quality, and reliability of the analysis. However, this review has significant limitations. The analysis was limited to studies published in English and Polish, which may have excluded important studies in other languages and limited the representativeness of the results in certain regions. Furthermore, focusing exclusively on the last 5 years may lead to the omission of historical trends or fundamental studies that provide a broader context. Another limitation may be the diversity of definitions and measurements of loneliness and social isolation in the studies analyzed.

## CONCLUSIONS

The level of loneliness and social isolation in old age is a complex issue. Retirement is ambiguously linked to an increased feeling of loneliness. Cultural aspects and social relationships outside the workplace also play an important role here. The results show that social ties appear to be the most important mitigating factors in preventing loneliness among older people [24]. Given the scale of loneliness among older people – estimated at around a quarter of the global senior population – and its documented negative impact on mental and physical health, there is an urgent need for coordinated action in the area of public health. These interventions should be aimed at reducing the level of perceived loneliness in this age group, constituting an important element of strategies promoting healthy aging, which is in line with the assumptions of the state's senior policy.

## References

[1] Sugiura K, Takase M, Isuzu N, Watanabe S, Murayama H. Relationship between employment status, loneliness, and social isolation: a systematic review. Res Square. 2024. 10.21203/rs.3.rs-5282848/v1.

[2] World Health Organization [Internet]. Geneva: WHO; 2025 [cited 2025 Oct 3]. Ageing. Available from: https://www.who.int/europe/health-topics/ageing#tab=tab_2.

[3] Red Cross Office Europe [Internet]. Brussels: Red cross [cited 2025 Oct 7]. Fighting loneliness. Available from: https://redcross.eu/projects/fighting-loneliness.

[4] Garattini L, Nobili A, Mannucci PM. Loneliness among older adults in Europe: time to integrate health and social care. Intern Emerg Med. 2025;20:639–42. 10.1007/s11739-025-03924-4. 40131638

[5] Perlman D, Peplau LA. Toward a social psychology of loneliness. In: Gilmour R, Duck S, editors. Personal relationships: relationships in disorder. London: Academic Press; 1981. p. 31–56.

[6] Townsend P. The family life of old people: an inquiry in East London. Harmondsworth (UK): Penguin Books; 1963.

[7] Galvez-Hernandez P, González-de Paz L, Muntaner C. Primary care-based interventions addressing social isolation and loneliness in older people: a scoping review. BMJ Open. 2022;12(2):e057729. 10.1136/bmjopen-2021-057729. PMC8819903 35121608

[8] Hansen T, Nes RB, Hynek K, Nilsen TS, Reneflot A, Stene-Larsen K, et al. Tackling social disconnection: an umbrella review of RCT-based interventions targeting social isolation and loneliness. BMC Public Health. 2024;24(1):1917. 10.1186/s12889-024-19396-8. 39020331 PMC11256365

[9] Russell D, Cutrona CE, Rose J, Yurko K. Social and emotional loneliness: an examination of Weiss's typology of loneliness. J Pers Soc Psychol. 1984;46(6):1313–21. 6737214 10.1037//0022-3514.46.6.1313

[10] Donovan NJ, Blazer DG. Social isolation and loneliness in older adults: review and commentary of a National Academies report. Am J Geriatr Psychiatry. 2020;28(12):1233–44. 10.1016/j.jagp.2020.08.005. 32919873 PMC7437541

[11] National Academies of Sciences, Engineering, and Medicine. Social Isolation and Loneliness in Older Adults: Opportunities for the Health Care System 2020. Waszyngton (DC): National Academies Press; 2020. 10.17226/25663. 32510896

[12] Greš A, Spasić N, Staver D. Loneliness in retirement. Medeni Med J. 2025;40(2):53–60. 10.4274/MMJ.galenos.2025.24804. 40569782 PMC12203447

[13] Guthmuller S, Heger D, Hollenbach J, Werbeck A. The impact of retirement on loneliness in Europe. Sci Rep. 2024;14(1):26971. 10.1038/s41598-024-74692-y. 39528537 PMC11555320

[14] Thompson C, Halcomb E, Masso M. The contribution of primary care practitioners to interventions reducing loneliness and social isolation in older people: an integrative review. Scand J Caring Sci. 2023;37(3):611–27. 10.1111/scs.13151. 36732897

[15] Kolk DV, Andringa G, de Korne DF, Huijsman R. A scoping review of influencing factors associated with loneliness in nursing home settings. Aging Ment Health. 2025;29:1–14. 10.1080/13607863.2025.2552427. 41017386

[16] Hajek A, Sutin AR, Posi G, Stephan Y, Peltzer K, Terracciano A, et al. Chronic loneliness and chronic social isolation among older adults: a systematic review, meta-analysis and meta-regression. Aging Ment Health. 2025;29(2):185–200. 10.1080/13607863.2024.2385448. 39126212

[17] Liu X, Yu HJ, Gao Y, Zhou J, Zhou M, Wan L, et al. Combined association of multiple chronic diseases and social isolation with functional disability after stroke in elderly patients: a multicenter cross-sectional study in China. BMC Geriatr. 2021;21:495. 10.1186/s12877-021-02439-9. 34530729 PMC8447675

[18] Gough C, Lewis LK, Barr C, Maeder A, George S. Community participation of community-dwelling older adults: a cross-sectional study. BMC Public Health. 2021;21(1):612. 10.1186/s12889-021-10592-4. 33781223 PMC8008662

[19] Fancourt D, Tymoszuk U. Cultural engagement and incident depression in older adults: evidence from the English longitudinal study of ageing. Br J Psychiatry. 2019;214(4):225–9. 10.1192/bjp.2018.267. 30560742 PMC6429253

[20] Hajek A, Zwar L, Gyasi RM, Yon DK, Pengpid S, Peltzer K, et al. Determinants of loneliness among older adults: a systematic review based on recent longitudinal studies. Arch Gerontol Geriatr. 2025;138:105953. 10.1016/j.archger.2025.105953. 40749605

[21] Jutengren G, Ståhl F. Determinants of social loneliness among older adults in job retirement and the role of emotional expressivity. Aging Ment Health. 2024;28(8):1153–61. 10.1080/13607863.2024.2338205. 38619317

[22] Björnwall A, Mattsson Sydner Y, Koochek A, Neuman N. Eating alone or together among community-living older people: a scoping review. Int J Environ Res Public Health. 2021;18(7):3495. 10.3390/ijerph18073495. 33801775 PMC8036467

[23] Balki E, Hayes N, Holland C. Effectiveness of technology interventions in addressing social isolation, connectedness, and loneliness in older adults: systematic umbrella review. JMIR Aging. 2022;5(4):e40125. 10.2196/40125. 36279155 PMC9641519

[24] Smale B, Wilson J, Akubueze N. Exploring the determinants and mitigating factors of loneliness among older adults. Wellbeing Space Soc. 2022;3:100089. 10.1016/j.wss.2022.100089.

[25] Juma F, Fernández-Sainz A, Vercauteren T, Stegen H, Häussermann F, De Donder L, et al. Intensity and changes in grandparental caregiving: exploring the link to loneliness in Europe. Arch Gerontol Geriatr. 2025;128:105630. 10.1016/j.archger.2024.105630. 39342889

[26] Hoang P, King JA, Moore S, Moore K, Reich K, Sidhu H, et al. Interventions associated with reduced loneliness and social isolation in older adults: a systematic review and meta-analysis. JAMA Netw Open. 2022;5(10):e2236676. 10.1001/jamanetworkopen.2022.36676. 36251294 PMC9577679

[27] Tcymbal A, Abu-Omar K, Hartung V, Bußkamp A, Comito C, Rossmann C, et al. Interventions simultaneously promoting social participation and physical activity in community living older adults: a systematic review. Front Public Health. 2022;10:1048496. 10.3389/fpubh.2022.1048496. 36568739 PMC9768837

[28] Tully MA, McMullan II, Blackburn NE, Wilson JJ, Coll-Planas L, Deidda M, et al. Is sedentary behavior or physical activity associated with loneliness in older adults? Results of the European-wide SITLESS study. J Aging Phys Act. 2020;28(4):549–55. 10.1123/japa.2019-0311. 31860832

[29] Pollak C, Verghese J, Blumen H. Loneliness and functional decline in aging: a systematic review. Res Gerontol Nurs. 2023;16(4):202–12. 10.3928/19404921-20230503-02. 37159388 PMC10926714

[30] Pollak C, Verghese J, Buchman AS, Jin Y, Blumen HM. Loneliness predicts progression of frailty in married and widowed, but not unmarried, community-dwelling older adults. J Frailty Aging. 2024;13(2):163–71. 10.14283/jfa.2024.27. 38616373 PMC11898203

[31] Nakou A, Dragioti E, Bastas NS, Zagorianakou N, Kakaidi V, Tsartsalis D, et al. Loneliness, social isolation, and living alone: a comprehensive systematic review, meta-analysis, and meta-regression of mortality risks in older adults. Aging Clin Exp Res. 2025;37(1):29. 10.1007/s40520-024-02925-1. 39836319 PMC11750934

[32] Schutter N, Holwerda TJ, Comijs HC, Stek ML, Peen J, Dekker JJM. Loneliness, social network size and mortality in older adults: a meta-analysis. Eur J Ageing. 2022;19(4):1057–76. 10.1007/s10433-022-00740-z. 36467548 PMC9685120

[33] Sipowicz K, Podlecka M, Mokros Ł, Pietras T. Lonely in the city – sociodempographic status and somatic morbidities as predictors of loneliness and depression among seniors: preliminary results. Int J Environ Res Public Health. 2021;18(14):7213. 10.3390/ijerph18147213. 34299666 PMC8305915

[34] Hounkpatin H, Simpson G, Santer M, Farmer A, Dambha-Miller H. Multiple long-term conditions, loneliness and social isolation: a scoping review of recent quantitative studies. Arch Gerontol Geriatr. 2024;120:105347. 10.1016/j.archger.2024.105347. 38309103

[35] Bevilacqua G, D'Angelo S, Ntani G, Syddall HE, Harris EC, Linaker C, et al. Older working adults in the HEAF study are more likely to report loneliness after two years of follow-up if they have negative perceptions of their work quality. BMC Public Health. 2021;21(1):574. 10.1186/s12889-021-10610-5. 33757464 PMC7988922

[36] Sutin AR, Luchetti M, Aschwanden D, Lee JH, Sesker AA, Stephan Y, et al. Sense of purpose in life and concurrent loneliness and risk of incident loneliness: an individual-participant meta-analysis of 135,227 individuals from 36 cohorts. J Affect Disord. 2022;309:211–20. 10.1016/j.jad.2022.04.084. 35483500 PMC9133197

[37] Resna RW, Widianti N, Nofiantoro W, Iskandar R, Ashbahna DM, Royani S, et al. Social environment support to overcome loneliness among older adults: a scoping review. Belitung Nurs J. 2022;8(3):197–203. 10.33546/bnj.2092. 37547116 PMC10401387

[38] Gallardo-Peralta LP, Sánchez-Moreno E, Rodríguez Rodríguez V, García Martín M. La investigación sobre soledad y redes de apoyo social en las personas mayores: una revisión sistemática en Europa. Rev Esp Salud Publica. 2023;97:e202301006. 36700292 PMC10540907

[39] Hreha K, Samper-Ternent R, Whitson HE, Downer LP, West JS, Downer B, et al. The association of vision and hearing impairment on cognitive function and loneliness: evidence from the Mexican health and aging study. J Aging Health. 2025;37(5–6):337–46. 10.1177/08982643241247583. 38621720 PMC11473705

[40] Qorbani S, Majdabadi ZA, Nikpeyma N, Haghani S, Shahrestanaki SK, Poortaghi S. The effect of participation in support groups on retirement syndrome in older adults. BMC Geriatr. 2024;24(1):333. 10.1186/s12877-024-04923-4. 38609838 PMC11010321

[41] Rodrigues PMF, Delerue-Matos A. The effect of social exclusion on the cognitive health of middle-aged and older adults: a systematic review. Arch Gerontol Geriatr. 2025;130:105730. 10.1016/j.archger.2024.105730. 39731813

[42] Salari N, Najafi H, Rasoulpoor S, Canbary Z, Heidarian P, Mohammadi M. The global prevalence and associated factors of loneliness in older adults: a systematic review and meta-analysis. Humanit Soc Sci Commun. 2025;12:985. 10.1057/s41599-025-05304-x.

[43] Robinson E, Daly M, Putra IGNE. The psychological burden associated with metabolic syndrome: evidence from UK and US older adults. Obes Sci Pract. 2024;10:e780. 10.1002/osp4.780. 38974477 PMC11227276

[44] Puyané M, Chabrera C, Camón E, Cabrera E. Uncovering the impact of loneliness in ageing populations: a comprehensive scoping review. BMC Geriatr. 2025;25:244. 10.1186/s12877-025-05846-4. 40211165 PMC11984289

[45] Band R, Rogers A. Understanding the meaning of loneliness and social engagement for the workings of a social network intervention connecting people to resources and valued activities. Health Expect. 2024;27(6):e70111. 10.1111/hex.70111. 39575523 PMC11582479

[46] Evans IEM, Llewellyn DJ, Matthews FE, Matthews FE, Woods RT, Brayne C, et al. Social isolation, cognitive reserve, and cognition in older people with depression and anxiety. Aging Ment Health. 2018;23(12):1691–701. 10.1080/13607863.2018.1506742. 30518250

[47] Lin Y, Zhu T, Zhang X, Zeng Z. Trends in the prevalence of social isolation among middle and older adults in China from 2011 to 2018: the China health and retirement longitudinal study. BMC Public Health. 2024;24:339. 10.1186/s12889-024-17734-4. 38302982 PMC10832184

[48] Hagani N, Clare PJ, Luo M, Merom D, Smith BJ, Ding D. Effect of retirement on loneliness: a longitudinal comparative analysis across Australia, China and the USA. J Epidemiol Community Health. 2024;78(10):602–8. 10.1136/jech-2023-221606. 38834231 PMC11420738

[49] Carr S, Fang C. A gradual separation from the world: a qualitative exploration of existential loneliness in old age. Ageing Soc. 2023;43(6):1436–56. 10.1017/S0144686X21001252. 38032719

[50] Sapkota A. Development of loneliness among older adults after retirement [Internet]. Oslo: OsloMet; 2020 [cited 2025 Oct 17]. Available from: https://oda.oslomet.no/oda-xmlui/bitstream/handle/11250/2754532/Sapkota_SIW_2020.pdf.

[51] Leung JC. Family support and community services for older adults in China: integration and partnership. In: Cheng S-T, editor. Handbook of Asian aging. New York: Routledge; 2018. p. 405–30.

[52] Zhang J, Fokkema T, Arpino B. Loneliness among Chinese older adults: the role of grandparenthood and grandparental childcare by gender. J Fam Issues. 2022;43:3078–99. 10.1177/0192513X211041992.

[53] Abramowska-Kmon A, Łątkowski W. The impact of retirement on happiness and loneliness in Poland: evidence from panel data. Int J Environ Res Public Health. 2021;18(18):9875. 10.3390/ijerph18189875. 34574798 PMC8465952

[54] Shin O, Park S, Lee H, Kang JY. Gender differences in the mechanism of involuntary retirement affecting loneliness through vulnerability and coping resources. Ageing Soc. 2024;44:1–22. 10.1017/S0144686X21001914.

[55] World Health Organization [Internet]. Geneva: WHO; 2025 [cited 2025 Oct 27]. In-person interventions for reducing social isolation and loneliness: Evidence and gap map. Available from: https://www.who.int/initiatives/decade-of-healthy-ageing/evidence-gap-map/sil-inperson.

